# Analysis of the achievement of primary and secondary goals and influencing factors in single-rooted immature permanent teeth after regenerative endodontic procedures: a retrospective study

**DOI:** 10.1186/s12903-023-03553-3

**Published:** 2023-11-11

**Authors:** Xijun Jiang, He Liu

**Affiliations:** Department of Pediatric Dentistry, Peking University School and Hospital of Stomatology & National Center for Stomatology & National Clinical Research Center for Oral Diseases & National Engineering Research Center of Oral Biomaterials and Digital Medical Devices & Beijing Key Laboratory of Digital Stomatology & Research Center of Engineering and Technology for Computerized Dentistry Ministry of Health & NMPA Key Laboratory for Dental Materials, Beijing, China

**Keywords:** Regenerative endodontic procedures, Primary goal, Secondary goal

## Abstract

**Objective:**

This study explored the achievement of primary and secondary goals and factors influencing their achievement in regenerative endodontic procedures (REPs) for immature permanent teeth.

**Methods:**

Dental records of all patients who received REPs for immature permanent teeth at the Department of Pediatric Dentistry, Peking University School and Hospital of Stomatology between January 2012 and January 2023 were retrieved. The evaluation of the primary goal was based on medical and radiographic records. The achievement of the primary goal was defined as the absence of clinical signs and symptoms, such as pain, swelling, or sinus tract and the absence of periapical radiolucency, as assessed on postoperative periapical radiographs. The achievement of secondary goal represented increased root wall thickness and/or increased root length, that is, continued root development. Periapical radiographs before and after treatment were used to evaluate the achievement of the secondary goal. The secondary goal was required to be achieved alongside the achievement of primary goal.

**Results:**

A total of 436 teeth (136 anterior and 300 posterior teeth) were included in this study, 96.1% of which demonstrated achievement of the primary goal. Signs of failure (17 teeth) included crown fracture and uncontrolled and recurrent periapical lesions. In addition, 77.8% of teeth demonstrated achievement of the secondary goal, and more than half of the teeth exhibited a complete root development. Evaluation factors included patients’ age, sex, tooth type, etiology, preoperative periapical lesion, duration of clinical signs and symptoms, follow-up period, and stage of root development. The achievement of the primary and secondary goals were significantly related to age and tooth type (p < 0.05).

**Conclusions:**

Children with a younger initial visit age are more likely to achieve primary and secondary goals. Additionally, posterior teeth had an advantage over anterior teeth in achieving primary and secondary goals.

## Background

Since the official application of regenerative endodontic procedures (REPs) into clinical practice, numerous prognostic studies have been conducted exploring its various aspects, including clinical success rate, qualitative and quantitative analysis of root development, and root canal calcification [1–5]. According to the “Clinical Considerations for a Regenerative Procedure” by the American Association of Endodontists (AAE), the degree of success of REPs is measured by the extent to which it is possible to attain primary, secondary, and tertiary goals [6]. The primary goal focuses on the elimination of symptoms and evidence of bony healing, which is also known as clinical success. Notably, different studies have reported varying clinical success rates over different time periods [7–9]. The secondary goal involves achieving increased root wall thickness and/or increased root length, that is, continued root development. The continued root development of immature permanent teeth after REPs is unpredictable. Studies have reported several patterns of root development in immature permanent teeth [3, 10]. If the root continues to develop, even without complete root formation, it can be considered a successful attainment of the secondary goal. The achievement of the secondary goal is valuable for improving fracture resistance and long-term prognosis of teeth. However, studies examining the achievement rate of secondary goals after REPs in immature permanent teeth are lacking. The tertiary goal represents a positive response to vitality testing, which, if achieved, could indicate a more organized vital pulp tissue. However, the results of the tertiary goal have been reported to be unreliable, which may be attributed to inconsistencies in positive sensibility results across studies, possibly due to difficulties in evaluating sensibility because of the presence of layered coronal fillings over the bioactive cement seal [11]. Therefore, the tertiary goal has a low reference value. For patients and doctors, achieving primary and secondary goals is more meaningful and practical for the determining long-term prognoses of teeth [12].

In the field of REPs for immature permanent teeth, there is a dearth of studies with a large sample size investigating the achievement rate of tertiary goals, principally primary and secondary goals, and the factors that affect their achievement. In this retrospective study, we aimed to summarize the achievement rate of different goals after REPs and analyze the factors that affect the achievement of these goals in immature permanent teeth. Since the tertiary goal is unreliable [11], and some teeth exhibited inconsistent pulp sensibility results at different follow-up times during the data-collecting process, the tertiary goal was excluded from the study analysis. The main purpose of this study was to explore the achievement of primary and secondary goals and the factors affecting their achievement in REPs through a large sample of cases.

## Methods

### Ethics

The study protocol was approved by the Ethics Committee of the Peking University School and Hospital of Stomatology (ref.PKUSSIRB-202,054,062). Dental records of all patients who received REPs from January 2012 to January 2023 for immature permanent teeth at the Department of Pediatric Dentistry, Peking University School, and Hospital of Stomatology were retrieved.

### Clinical protocol

All patients and caregivers were provided with a comprehensive explanation about REPs. Comprehensive discussions on the associated risks, complications, and alternative treatment options were conducted, and parental consent was obtained before treatment.

All the procedures were performed following the recommended protocol by the AAE [6] at the time of treatment. Any variations in the protocol were attributed to the revision of the AAE guidelines in lieu of emerging evidence over the study period from 2012 to 2023. During the first appointment, a preoperative periapical radiograph was acquired, and the teeth were accessed under rubber dam isolation. Subsequently, the teeth were disinfected using 1.5–3% NaOCl (20 mL/canal, 5 min) or a combination of NaOCl (20 mL/canal, 5 min) and saline (20 mL/canal, 5 min). Calcium hydroxide paste or a low-concentration (1 g/mL) antibiotic paste (triple /double antibiotic paste) was placed as an intracanal medicament, and the access was sealed using a temporary restorative material (glass ionomer) for 2–4 weeks. During the second appointment, the teeth were anesthetized using lidocaine (2%) without a vasoconstrictor, re-accessed, and irrigated with a final rinse of 20 mL of 17% ethylenediaminetetraacetic acid (EDTA) and/or 17% EDTA followed by saline. A pre-curved K-file was overextended 2 mm apically to induce bleeding inside the canal. Various types of scaffolds were applied, including blood clots, platelet-rich fibrin, and collagen membranes [3]. Teeth were then restored with a capping material (i.e., ProRoot mineral trioxide aggregate [MTA, Dentsply Tulsa Dental, Johnson City, TN, USA], iRoot BP [Innovative Bioceramix Inc. Canada], or Fuji IX GIC [Fuji Corporation, Osaka, Japan]) followed by Z250/P60 composite resin (3 M ESPE; Irvine, California; 3–4 mm). A postoperative periapical radiograph was acquired, and all patients were evaluated at 3-month intervals.

### Patient selection

Children who underwent REPs in the Department of Pediatric Dentistry, Peking University School and Hospital of Stomatology, Beijing, China, between January 2012 and January 2023 were enrolled. The inclusion criteria were as follows: (1) single-rooted immature permanent teeth; and (2) complete medical and radiographic records. Teeth that demonstrated failure in achieving complete root formation were required to undergo follow up for at least 6 months. The follow-up time was not limited to the teeth that achieved complete root formation.

### Evaluation of treatment outcomes

Evaluation factors included patients’ age, sex, tooth type, etiology, preoperative periapical lesion, duration of clinical signs and symptoms, follow-up period, and stage of root development [13].

The primary goal was evaluated based on medical and radiographic records. The achievement of the primary goal was defined as the absence of clinical signs and symptoms, such as pain, swelling, or a sinus tract and the absence of periapical radiolucency as assessed on postoperative periapical radiographs. Conversely, failure was determined by the presence of clinical signs or symptoms or the persistence or recurrence of periapical radiolucency and/or tooth fracture. Uncontrolled periapical lesion referred to the persistent presence of periapical radiolucency throughout the follow-up process, without obvious resolution of the periapical lesion or healing of the periapical bone, as assessed on postoperative periapical radiographs. Recurrent periapical lesions referred to the absence of clinical symptoms and periapical radiolucency after treatment, followed by reappearance of the periapical lesion during the long-term follow up. Tooth fracture referred to the breaking and detachment of the crown, leaving only the root in the alveolar bone.

The secondary goal was evaluated based on periapical radiographs before and after treatment. Based on previous studies [3, 10], three types of root development patterns were considered indicative of continued root development. Type I involved increased thickening of the canal walls and continued root maturation. Type II involved increased thickening of the canal walls without a significant increase in root length, resulting in blunt and closed root apices. Type III involved continued root development with an open apical foramen. In contrast, other types of root development patterns were considered a failure to achieve the secondary goal. The evaluation of the secondary goal was performed in a blinded manner by two independent reviewers, both of whom were researchers involved in the study. A consensus was reached through discussion when an evaluation was not unanimous.

The secondary goal was required to be achieved alongside the achievement of primary goal. In this study, several teeth exhibited continued root development; however, when apical lesions remained uncontrolled or exhibited recurrence, these teeth were considered failed cases and were not considered as having achieved the secondary goal.

### Statistical analysis

The collected data were converted into a Microsoft Excel (Microsoft, Redmond, WA) spreadsheet and then imported into the Statistical Package for the Social Sciences software (version 20; IBM, Armonk, NY) for further analyses. Logistic regression analysis was performed.

## Results

**Fig. 1:**
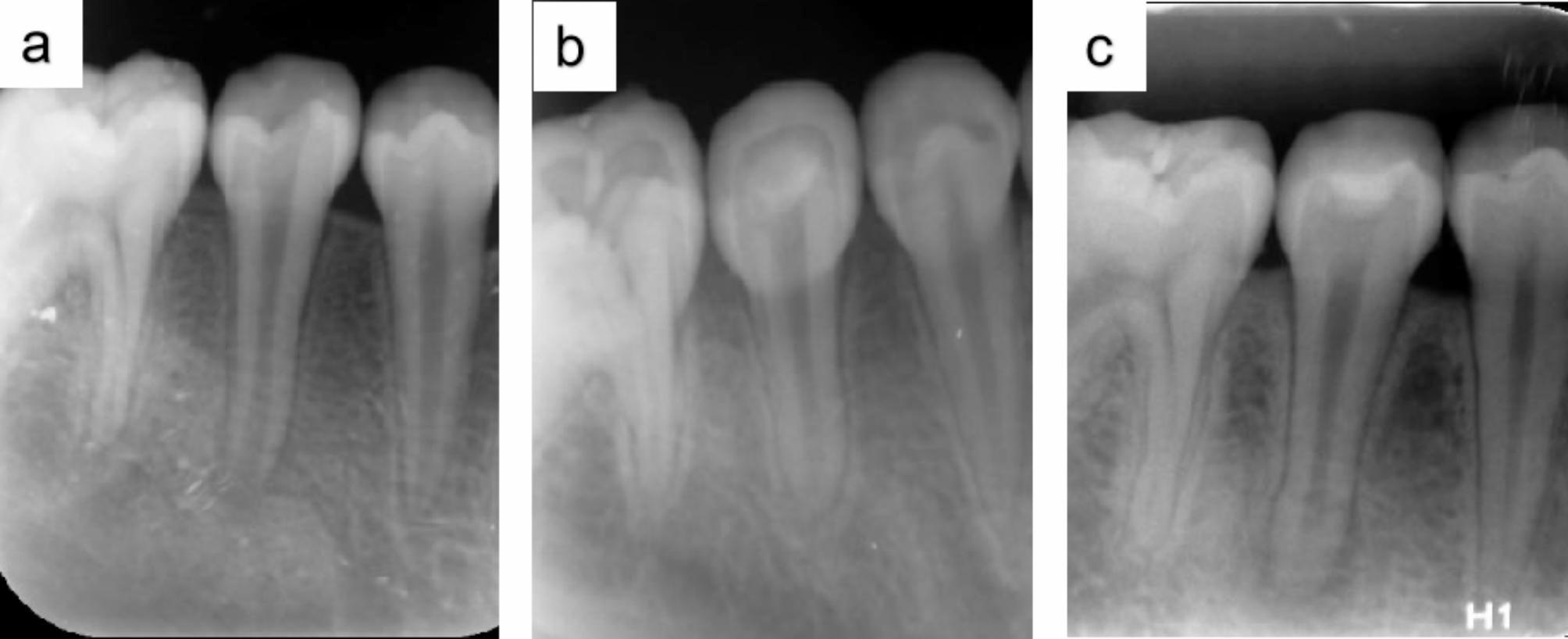
Periapical radiographs over the 60-month follow-up period. (**a**) Preoperative periapical radiograph of tooth 45 showing an incompletely developed root with a periapical lesion. (**b**) Postoperative periapical radiograph at the 18-month follow-up period after REPs showing resolution of the periapical lesion and healing of the periapical bone, with complete development of the root. (**c**) Postoperative periapical radiograph at the 60-month follow-up period showing a recurrence of the periapical lesion while the patient remained asymptomatic

**Fig. 2:**
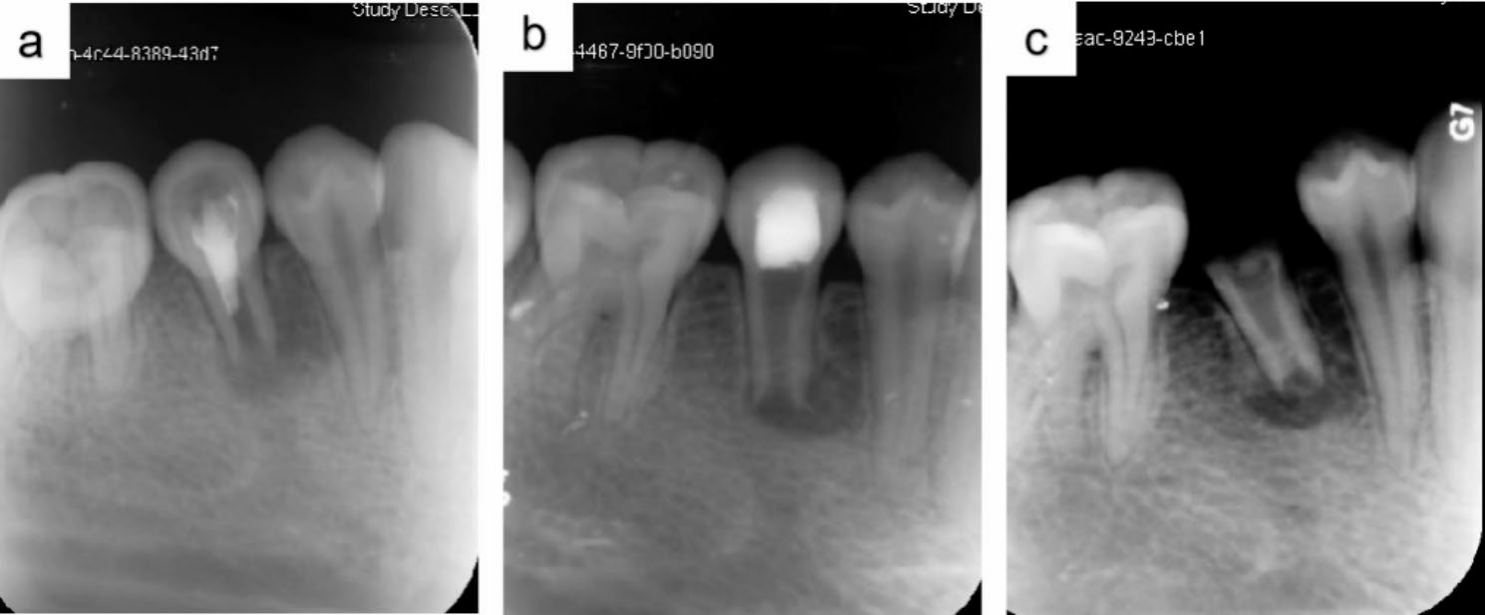
Periapical radiographs over the 16-month follow-up period. (**a**) Periapical radiograph of tooth 45 showing an incompletely developed root with a periapical lesion. (**b**) Postoperative periapical radiograph at the 9-month follow-up period showing no obvious resolution of the periapical lesion or healing of the periapical bone. (**c**) Postoperative periapical radiograph at the 16-month follow-up period showing sustained periapical lesion and crown fracture

**Fig. 3:**
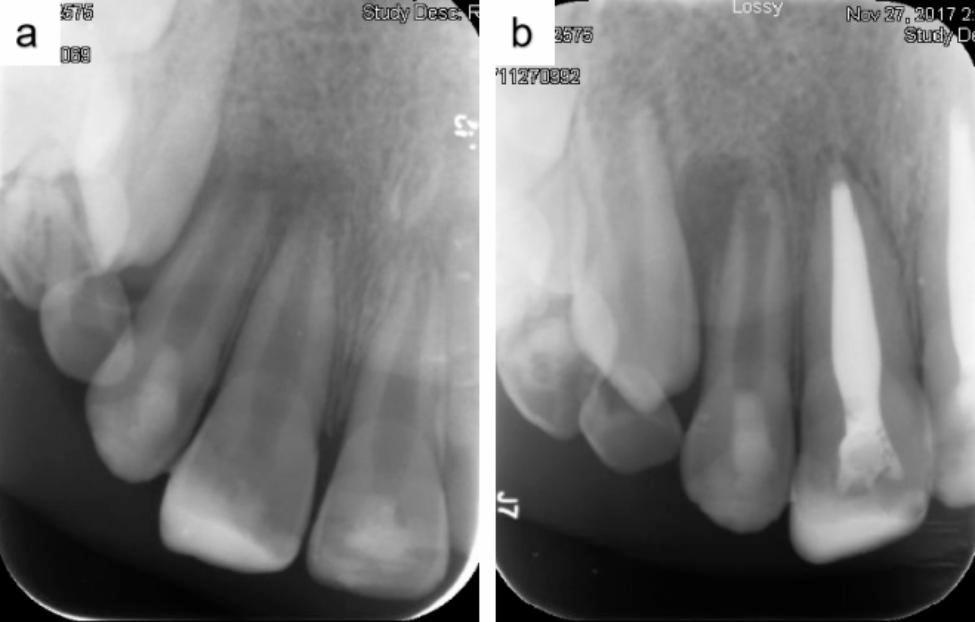
Periapical radiographs over the 9-month follow-up period. (**a**) Postoperative periapical radiograph of tooth 12 showing an incompletely developed root with a periapical lesion. (**b**) Postoperative periapical radiograph at the 15-month follow-up showing sustained periapical lesion even though a complete root development is achieved

We included 436 teeth (136 anterior and 300 posterior teeth) from 408 patients (212 males and 196 females), aged 6–16 years (average 10.17 ± 1.82 years). The average follow-up time was 26.11 ± 19.95 months (range: 4–107 months). Among the 436 teeth, 419 demonstrated achievement of the primary goal. However, 17 teeth failed to demonstrate achievement of the primary goal, presenting with persistence or a recurrence of periapical radiolucency or the need for tooth extraction because of tooth fracture (Figs. 1, 2 and 3). In addition, 339 teeth exhibited continued root development, with the achievement rate of the secondary goal being 77.8%. Of the 339 teeth, 261 completed root development (Table 1).

**Table 1:** Demographics and clinical characteristics of the study participants

Variables	
Age (years)	
Range Mean ± SD	6–16 10.17 ± 1.82
Sex, (*n*)	
Male Female	212 196
Tooth type, (*n*)	
Anterior Premolar	136 300
Follow-up period (months)	
Range Mean ± SD	4–107 26.11 ± 19.95
Primary goal, *n* (*%*)	
Achieved Not achieved	419 (96.1) 17 (3.9)
Secondary goal, *n* (*%*)	
Achieved Root formation Not achieved	339 (77.8) 261 97 (22.2)

In this study, 96.1% of teeth demonstrated achievement of the primary goal. A statistical analysis was conducted to investigate the influence of different factors on the achievement of the primary goal and was observed that sex, etiology, root development stage, preoperative periapical lesions, and duration of symptoms did not significantly affect the achievement of the primary goal. However, a significant difference in age was noted between children who achieved and those who did not achieve the primary goal. The average age of children who achieved the primary goal was 10.13 ± 1.77 years, whereas that of children who did not achieve the primary goal was 11.24 ± 2.66 years. Children with a younger initial visit age were more likely to achieve the primary goal. The type of teeth also exhibited an impact on achieving the primary goal; posterior teeth were more likely to demonstrate achievement of the primary goal than anterior teeth (Table 2).

**Table 2:** Analysis of the achievement of primary goal

Variables	Achieved primary goal	Not achieved a primary goal	P-value ^a^
Age, (years)			<0.05 (OR 0.53)
Mean ± SD	10.13 ± 1.77	11.24 ± 2.66	
Sex, (*n*)			0.538
Male Female	202 190	11 6	
Tooth type, (*n*)			<0.05 (OR 26.41)
Anterior Premolar	126 293	10 7	
Etiologies, (*n*)			
Trauma Dens evaginatus Caries	113 299 7	8 8 1	0.762 0.564 0.959
Stage of root development ^c^, (*n*)			
6–7 7 8 9	2 39 280 98	0 0 11 6	0.958 0.999 0.998 0.578
Preoperative periapical lesion, (*n*)			0.998
Without With	33 386	0 17	
Duration of clinical symptoms (days)			0.520
Mean ± SD	16.58 ± 36.04	20.53 ± 33.41	
Follow-up period (months)			0.836
Mean ± SD	25.98 ± 19.90	29.24 ± 21.53	

SD, standard deviation

^a^Logistic regression analysis

^b^Nolla’s stage of root development

In this study, 77.8% of teeth demonstrated achievement of the secondary goal, including three types of root development patterns: types I, II, and III, as described earlier. Type I, increased thickening of the canal walls and continued root maturation; type II, increased thickening of the canal walls without a significant increase in root length, with the root apex becoming blunt and closed; type III, continued root development with the apical foramen remaining open. Statistical analysis revealed that sex, etiology, root development stage, preoperative periapical lesions and symptom duration had no significant impact on the achievement of the secondary goal. However, the achievement of the secondary goal was significantly related to age and tooth type. Children with a younger initial visit age were more likely to achieve the secondary goal. The type of teeth also exhibited an impact on achieving the secondary goal; posterior teeth were more likely to demonstrate achievement of the secondary goal than anterior teeth (Table 3).

**Table 3:** Analysis of the achievement of secondary goal

Variables	Achieved secondary goal	Not achieved secondary goal	*P*-value ^a^
Age, (years)			<0.05 (OR 0.83)
Mean ± SD	10.18 ± 1.73	10.14 ± 2.11	
Sex, (*n*)			0.978
Male Female	177 162	54 43	
Tooth type, (*n*)			<0.05 (OR 4.29)
Anterior Premolar	92 247	44 53	
Etiologies, (*n*)			
Trauma Dens evaginatus Caries	83 251 5	38 56 3	0.943 0.800 0.996
Stage of root development ^c^, (*n*)			
6–7 7 8 9	2 31 223 83	0 8 68 21	0.686 0.999 0.374 0.249
Preoperative periapical lesion, (*n*)			0.089
Without With	30 309	3 94	
Duration of clinical symptoms (days)			0.689
Mean ± SD	17.06 ± 37.68	15.60 ± 29.04	
Follow-up period (months)			0.191
Mean ± SD	26.41 ± 20.15	25.05 ± 19.29	

SD, standard deviation

^a^Logistic regression analysis

^b^Nolla’s stage of root development

## Discussion

This retrospective study included 436 immature permanent teeth, comprising 136 anterior and 300 posterior teeth, which underwent REPs over an 11-year period. Among them, 403 teeth had apical lesions at the first visit. The longest duration of the presence of apical lesions in the tooth was more than 1 year. After treatment, 96.1% of teeth demonstrated achievement of clinical success, with only a small proportion (approximately 3.9%) failing to do so, primarily manifesting as recurrent periapical lesions. Some of these lesions were detected through periapical radiographs, even when the patients remained asymptomatic (Fig. 1). This suggested the importance of regular follow up in detecting the recurrence of inflammation, despite the probability of such an occurrence is relatively low. The achievement rate of the secondary goal was 77.8%, and approximately 60% of the teeth exhibited complete root development. Most immature permanent teeth exhibited varying degrees of root development after REPs.

The achievement of the primary goal directly affected the survival of teeth. The success was closely related to the effectiveness of chemical disinfection of root canals. The difficulty at this stage lies in protecting the tissue around the apical area instead of focusing solely on eliminating the bacteria in the root canal. Some studies have indicated that current root canal disinfection measures and drug concentrations are insufficient to eliminate bacteria [14, 15], as some cases in this study demonstrated unsuccessful management of apical inflammation from the beginning, suggesting that residual bacteria might still be present. To protect the vitality of the dental papilla, we need not excessively pursue thorough root canal disinfection but rather maintain a balance between root canal disinfection and protection of the apical tissue. The high achievement rate of the primary goal in this study indicated that the current method of root canal disinfection effectively controlled periapical inflammation. The root canal sealing material is a second factor affecting the achievement of the primary goal [16]. The root canal sealing materials used in this study were mainly MTA and iRoot BP, and their good sealing and biocompatibility contributed to a high clinical success rate over an average follow-up period of more than 2 years. Furthermore, experienced clinicians played a vital role in determining the timing of inducing bleeding within the root canal during treatment, adherence to the treatment protocol, and frequency of chemical disinfection of root canals. In this study, the experienced clinicians treated all cases according to the AAE guidelines and optimized the treatment plan continuously according to changes in the guidelines, which was a critical factor contributing to the high clinical success rate.

The achievement rate of the secondary goal in this study was 77.8%; most teeth demonstrated varying degrees of root development after treatment. Some studies have suggested that the hard tissue formed in the root canal after REPs primarily resembles bone- and cementum-like tissue [17]; we still have a long journey ahead to regenerate dentin-like tissue. However, regardless of whether the newly formed hard tissue is dentin-like or not, the deposition of hard tissue holds great significance in strengthening the fracture resistance and improving the long-term prognosis of teeth, especially in teeth with poor initial root development conditions. From the researchers’ perspective, we hope to control the type of newly formed tissue; however, from a patient-centered consideration [12], patients can benefit as long as the root continues to develop and fracture resistance increases, regardless of the tissue resembling dentin-like tissue.

Regarding the impact of different factors on the achievement of the primary goal, the data in this study suggested that children with a younger initial visit age were more likely to achieve the primary goal. Additionally, posterior teeth exhibited an advantage over anterior teeth in achieving the primary goal. However, considering the limited number of teeth that exhibited failure to achieve the primary goal, the influence of age and tooth type on the achievement rate of the primary goal remains more of a reference, and further verification is needed. From the perspective of scientific research, these findings are unexpected and interesting, warranting further validation. Clinically, decision-making should neither be influenced by the age of children nor the type of teeth.

Regarding the impact of different factors on the achievement of the secondary goal, a study reported a relationship between age and root development. Compared to children aged 14–18 years, children aged 9–13 years demonstrated a significant progressive increase in root length and width and a decrease in apical diameter [18]. The age range of children included in this study was 6–16 years, and children with a younger initial visit age were more likely to achieve the secondary goal. The type of teeth also exhibited an impact on achieving the secondary goal; posterior teeth were more likely to demonstrate achievement of the secondary goal than anterior teeth. Another study reported that the expected outcome of radiographical root development is less predictable when immature permanent teeth with periradicular pathosis are treated with REPs [19]. The apical papilla exhibits a certain level of tolerance to apical lesions [20, 21], which serves as the physiological basis for continued root development after REPs in teeth with apical lesions. Of all the 436 teeth in this study, 403 (92.4%) had apical lesions at the first visit, and statistical analysis did not find a significant impact of apical lesions on the achievement rate of the secondary goal. In this study, the achievement of the secondary goal was significantly related to age and tooth type. Children with a younger initial visit age and posterior teeth are more likely to achieve secondary goals.

In a study analyzing 111 teeth after REPs, 16 teeth exhibited failure, with 56% of the failure attributed to trauma, 25% attributed to dens evaginatus, and 12.5% attributed to caries. The reasons for the failure were sustained infection (81.3%) and root resorption (18.7%) [22]. In the present study, 17 teeth exhibited failure, with 47% of the failure attributed to trauma, 47% attributed to dens evaginatus, and 6% attributed to caries. The signs of failure included crown fracture (24%), uncontrolled (29%), and recurrent periapical lesions (53%). One tooth exhibited failure to control periapical lesion and underwent crown fracture (Fig. 2). Infection was the main factor for failure, highlighting the necessity of regular follow ups after REPs. From the perspective of failed teeth that continued root development, five teeth exhibited failure to control apical lesions, of which one tooth completed root development (Fig. 3). Similar cases have been reported previously [23]. Nine teeth in this study experienced recurrent apical lesions, of which two teeth had recurrent apical lesions after root formation. However, even if these teeth continued root development, they were not considered to have achieved the secondary goal. The secondary goal was essentially considered to be achieved alongside the primary goal for it to be meaningful.

The limitation of this study was that intracanal medicaments were not analyzed as evaluation factors. It was reported that the occurrence of calcification after REPs is related to the use of calcium hydroxide paste [24]. A higher percentage of root dentin wall thickening was reported with the use of antibiotic pastes as an intracanal medicament, whereas a higher percentage of apical closure was observed when calcium hydroxide was used [25]. In the present study, the information on intracanal medicaments in each case was obtained from medical records, and many cases involved cross-application of these two medicaments, thus making it an unsuitable evaluation factor. Further targeted clinical and basic studies are needed to confirm the observed differences and explore the underlying reasons to provide valuable insights for endodontists when planning REPs.

## Conclusions

Immature permanent teeth can demonstrate a remarkably high rate of clinical success (primary goal) after REPs based on the current AAE protocol. Regular follow-ups should also be conducted to detect the recurrence of inflammation, even when the teeth remain asymptomatic. Most teeth could achieve varying degrees of root development after treatment (secondary goal), and more than half of the teeth could achieve complete root formation. Children who were younger at initial visit age were more likely to achieve primary and secondary goals. Additionally, posterior teeth had an advantage over anterior teeth in achieving primary and secondary goals.

## Data Availability

The datasets generated and analyzed during the current study are not publicly available due to individual privacy but are available from the corresponding author upon reasonable request.
